# Incidence and severity of pertussis hospitalisations in infants aged less than 1 year in 37 hospitals of six EU/EEA countries, results of PERTINENT sentinel pilot surveillance system, December 2015 to December 2018

**DOI:** 10.2807/1560-7917.ES.2021.26.4.1900762

**Published:** 2021-01-28

**Authors:** Lore Merdrignac, Fatima Aït El Belghiti, Elisabetta Pandolfi, Mireia Jané, Jane Murphy, Kateřina Fabiánová, Manuel García Cenoz, Elmira Flem, Sophie Guillot, Alberto E Tozzi, Gloria Carmona, Adele Habington, Jana Zavadilová, Ana Navasués, Håkon Bøås, Daniel Lévy-Brühl, Beatrice Ferretti, Miguel Lanaspa, Niam O’Sullivan, Pavla Křížová, Leticia Fernandino, Terese Bekkevold, Thomas Hanslik, Carmen Muñoz-Almagro, Sabrina Bacci, Gianfranco Spiteri, Marta Valenciano, Alain Moren, PERTINENT Group, PERTINENT group, PERTINENT group, Lore Merdrignac, Camelia Savulescu, Marta Valenciano, Alain Moren, Pavla Křížová, Kateřina Fabiánová, Jana Zavadilová, Zuzana Blechová, Květa Bláhová, Pavel Kosina, Josef Sýkora, Alena Holčíková, Petr Širůček, Daniel Lévy-Brühl, Fatima Aït El Belghiti, Adèle Grembombo, Sophie Guillot, Sylvain Brisse, Julie Toubiana, Suzanne Cotter, Jane Murphy, Robert Cunney, Norma O’Shaughnessy, Adele Habington, Niamh O’Sullivan, Elisabetta Pandolfi, Alberto E Tozzi, Caterina Rizzo, Luisa Russo, Ilaria Campagna, Francesco Gesualdo, Sara Ciampini, Valentina Annarosa Ferro, Elena Boccuzzi, Elmira Flem, Håkon Bøås, Terese Bekkevold, Liliana Vazquez Fernandez, Carmen Muñoz-Almagro, Cristina Esteva, Miguel Lanaspa Perez, Mireia Jané, Gloria Carmona, Lesly Acosta, Yolanda Jordan Garcia, Manuel García Cenoz, Ana Navascués, Leticia Fernandino Zubieta, Jesús Castilla, Thomas Hanslik, Sabrina Bacci, Gianfranco Spiteri

**Affiliations:** 1 Epidemiology Department, Epiconcept, Paris, France; 2 Direction des maladies infectieuses, Santé Publique France, Paris, France; 3 Multifactorial Disease and Complex Phenotype Research Area, Bambino Gesù Children Hospital, Rome, Italy; 4 Epidemiological surveillance and response, Public Health Agency of Catalonia, Barcelona, Spain; 5 Research, Temple Street Children’s University Hospital, Dublin, Ireland; 6 National Institute of Public Health, Prague, Czech Republic; 7 Instituto de Salud Pública de Navarra, IdiSNA - Navarre Institute for Health Research, Pamplona, Spain; 8 Infectious Disease Epidemiology and Modeling, Norwegian Institute of Public Health, Oslo, Norway; 9 Biodiversité et Epidémiologie des bactéries et pathogènes, Institut Pasteur, Paris, France; 10 Chief Innovation Unit and Clinical Trials, Bambino Gesù Children Hospital, Rome, Italy; 11 Microbiology, Our Lady’s Children’s hospital Crumlin, Dublin, Ireland; 12 Clinical Microbiology Service, Complejo Hospitalario de Navarra, Pamplona, Spain; 13 Instituto de Recerca Pediatrica Hospital Sant Joan de Deu, Barcelona, Spain; 14 Sorbonne University, INSERM, Institut Pierre Louis d’Epidémiologie et de Santé Publique, Paris, France; 15 Universitat Internacional de Catalunya, Barcelona, Spain; 16 CIBER of Epidemiology and Public Health CIBERESP, Barcelona, Spain; 17 European Centre for Diseases Prevention and Control, Stockholm, Sweden; 18 The members of the network are listed at the end of the article

**Keywords:** pertussis incidence, active surveillance, hospital surveillance, pertussis

## Abstract

**Introduction:**

PERTINENT is a pilot active surveillance system of infants hospitalised with pertussis in six European Union/European Economic Area countries (37 hospitals, seven sites).

**Aim:**

This observational study aimed to estimate annual pertussis incidence per site from 2016 to 2018 and respective trends between 2017 and 2018. Pertussis cases were described, including their severity.

**Methods:**

We developed a generic protocol and laboratory guidelines to harmonise practices across sites. Cases were hospitalised infants testing positive for *Bordetella pertussis* by PCR or culture. Sites collected demographic, clinical, laboratory data, vaccination status, and risk/protective factors. We estimated sites’ annual incidences by dividing case numbers by the catchment populations.

**Results:**

From December 2015 to December 2018, we identified 469 cases (247 males; 53%). The median age, birthweight and gestational age were 2.5 months (range: 0–11.6; interquartile range (IQR): 2.5), 3,280 g (range: 700–4,925; IQR: 720) and 39 weeks (range: 25–42; IQR: 2), respectively. Thirty cases (6%) had atypical presentation either with cough or cyanosis only or with absence of pertussis-like symptoms. Of 330 cases with information, 83 (25%) were admitted to intensive care units including five deceased infants too young to be vaccinated. Incidence rate ratios between 2018 and 2017 were 1.43 in Czech Republic (p = 0.468), 0.25 in Catalonia (p = 0.002), 0.71 in France (p = 0.034), 0.14 in Ireland (p = 0.002), 0.63 in Italy (p = 0.053), 0.21 in Navarra (p = 0.148) and zero in Norway.

**Conclusions:**

Incidence appeared to decrease between 2017 and 2018 in all but one site. Enhanced surveillance of hospitalised pertussis in Europe is essential to monitor pertussis epidemiology and disease burden.

## Introduction

Most severe cases of pertussis (whooping cough) occur below 5 years of age. Worldwide, it causes substantial mortality in infants (85,900 estimated deaths in 2014) [1]. Sixty-three percent of cases aged less than 1 year reported to the European Centre for Disease Prevention and Control (ECDC) in 2017 required hospitalisation [2]. Severe complications include pneumonia, seizures, encephalopathy and death.

In neonatal infection, the cough with the characteristic whoop might be absent. The initial finding for *Bordetella pertussis* infection is frequently apnoea [3]. Since infants and neonates often have a clinical presentation that is atypical or similar to several other respiratory diseases, Vittuci et al. support a routine pertussis laboratory diagnosis in all infants aged less than 3 months with acute respiratory symptoms [4]. Adolescents (≥ 11 years of age) and adults (≥ 18 years of age) can develop a mild, often undiagnosed, form of the disease and represent a reservoir of transmission for infants.

Pertussis is one of the least controlled vaccine-preventable diseases in European Union/European Economic Area (EU/EEA) countries [5]. The number of pertussis cases reported to ECDC increased since 2011 despite a primary series of acellular pertussis vaccine coverage exceeding 90% in most countries [6]. The last peak incidence year occurred in 2012 with 42,500 reported cases, impacting 19 of 28 countries with different magnitude [5]. It affected adolescents, adults and particularly children too young to be vaccinated or to have completed the primary series. This increase may be explained by improved diagnostic methods, increased disease awareness, waning of acellular-vaccine-induced protection, or a lower vaccine effectiveness (VE) due to bacterial mutation [7].

At EU/EEA level, the routine pertussis surveillance systems are heterogeneous. Under-diagnosis and under-notification also differ across EU/EEA countries [8]. Even though a majority uses the EU case definition [9,10], differences in laboratory procedures, completeness of reporting and differences in disease awareness still remain [8]. Most case definitions do not distinguish between all *Bordetella* species that can have similar respiratory presentation and include indifferently *B. pertussis*, *B. parapertussis*, *B. holmesii* and *B. bronchiseptica*. *Bordetella* species may not have the same pathogenicity and may not be all directly targeted by pertussis-containing vaccine [3]. Therefore, existing surveillance systems make comparison of pertussis immunisation strategies and estimates of pertussis incidence at EU/EEA level difficult. Following the 2012 outbreak, an ECDC consultation resulted in a recommendation to conduct studies in the EU/EEA to measure the burden of pertussis including infant hospitalisations [11]. In recent years, some countries have enhanced surveillance of pertussis at hospital [12-14] or population level [15].

In 2015, ECDC initiated PERTINENT, ‘Pertussis in Infants European Network’, a hospital-based active pilot surveillance system in seven study sites using the same pertussis surveillance protocol. The surveillance system has two main objectives. The first is to identify the trends in incidence of laboratory-confirmed pertussis in hospitalised infants (≤1 year old) for the respective study sites. The second is to estimate VE using the test-negative design. In the current study, annual incidences per site are estimated from 2016 to 2018 as well as the change in incidence in 2018 compared to 2017. Pertussis cases are described by severity and other characteristics. VE results are not presented as VE will be assessed once the required sample size is reached.

## Methods

### Study design and setting

The PERTINENT coordination selected seven study sites (Czech Republic; France; Ireland; Italy; Norway; Catalonia, Spain and Navarra, Spain) willing to participate and able to comply with the generic PERTINENT sentinel surveillance protocol [16] and laboratory guidelines [17] and to estimate the participating hospitals’ catchment population. We organised site visits and a laboratory workshop to ensure the harmonisation of site-specific protocols allowing pooling of sites’ data.

From December 2015 to September 2016, study sites progressively implemented active surveillance in 41 hospitals. A large proportion of hospitals were situated in France (n = 21 hospitals) and other countries had one to six participating hospitals. Each site complied with the local ethical procedures. In May 2018, the number of participating hospitals was reduced to 37 after withdrawal of four Norwegian hospitals (Table 1). All sites use the acellular pertussis vaccine for the primary series in infants, but national vaccine recommendations and primary schedules vary across sites (Table 1 and supplementary Table S4).

**Table 1:** Characteristics of PERTINENT study sites, number of cases positive to *Bordetella* species and annual incidence by study site and year, Europe, 1 December 2015–31 December 2018 (n = 41 sites)

Study sites		Czech Republic	France	Ireland	Italy	Spain, Catalonia	Spain, Navarra	Norway
Vaccination recommendations: year introduction and doses recommended								
Primary schedule	Year of introduction	2018^a^	2013	1995	1995	2016^b^	2016^b^	1998
	Ages for different doses	3, 5, 11–13 months	2, 4, 11 months	2, 4, 6 months	3, 5, 11 months	2, 4, 11 months	2, 4, 11 months	3, 5, 12 months
Pregnancy, year of introduction		2016	No	2013	2017	2014	2015	No
Cocooning, year of introduction		No	2004	2013	No	No	No	No
Participating hospitals and catchment population estimation								
Number of hospitals participating in PERTINENT		6	21	2	2	1	4	5 *(2016–2018/05)* 1 *(from 2018/05)*
Method used to estimate hospital catchment population		National census	National census × estimation of PERTINENT hospital coverage [13]	National census	Regional census	National census × estimation of PERTINENT hospital coverage	National census	Regional census prorata temporis
Number of screened infants and cases per *Bordetella* species								
Screened infants in PERTINENT		73	546	138	509	207	118	523
*Bordetella pertussis *cases^c^		25	199^d^	30	145^e^	50	13	7
*Bordetella parapertussis *cases		0	10^d^	2	NA	1	4	NA
*Bordetella holmesii *cases		0	2	0	NA	1	1	NA
Other *Bordetella* species^f^		0	16	0	NA	3	0	NA
Total *Bordetella pertussis* cases and incidence, by year of study								
2016								
Number of cases 2016		8	45	6	61	19	7	4
Catchment population 2016		65,638	190,077	25,110	34,428	12,138	5,875	25,545
Incidence per 100,000 infants January 2016–December 2016		12.2	29.1^g^	57.2^g^	211.9^g^	156.5	119.1	17.1^g^
95% exact confidence interval		(5.3–24.0)	(21.2–38.9)	(21.0–124.4)	(162.1–272.1)	(94.3–244.3)	(47.9–245.3)	(4.7–43.8)
2017								
Number of cases 2017		7	91	21	47	25	5	3
Catchment population 2017		68,128	185,420	23,267	33,811	12,056	5,856	25,479
Incidence per 100,000 infants January 2017–December 2017		10.3	49.1	90.3	139.0	207.4	85.4	11.8
95% exact confidence interval		(4.1–21.2)	(39.5–60.3)	(55.9–137.9)	(102.2–184.8)	(134.2–306.0)	(27.7–199.1)	(2.4–34.4)
2018								
Number of cases 2018		10	63	3	28	6	1	0
Catchment population 2018		68,061	181,481	23,191	31,953	11,593	5,708	14,308^h^
Incidence per 100,000 infants January 2018–December 2018		14.7	34.7	12.9	87.6	51.8	17.5	0.0
95% exact confidence interval^i^		(7.0–27.0)	(26.7–44.4)	(2.7–37.8)	(58.2–126.6)	(19.0–112.6)	(0.4–97.6)	(0 –25.8)

NA: not available; PERTINENT: Pertussis in Infants European Network.

^a^ Before 2018: doses at 2, 3, 4 and 10 months.

^b^ Before 2016: doses at 2, 4 and 6 months.

^c^ Percentage of positivity among screened infants is expected to vary across sites due to the variation of hospitalisation likelihood of infants with pertussis-like symptoms.

^d^ Including one co-infection of *Bordetella pertussis* and *Bordetella parapertussis.*

^e^ Including nine cases recruited in 2015.

^f^ Laboratory could not confirm and differentiate the *Bordetella* species.

^g^ Incidence calculated *prorata temporis* based on available data in 2016 due to a progressive implementation of the surveillance.

^h^ Withdrawal of four hospitals.

^i^ ‘Exact confidence interval’ for incidence as the exact binomial confidence interval (i.e. Clopper–Pearson interval) was computed.

### Case identification and recruitment

The study population consisted of all infants aged less than 1 year, likely to be hospitalised in one of the participating hospitals if developing pertussis-like symptoms.

To maximise the sensitivity of the surveillance, we raised hospital physicians’ awareness of pertussis clinical presentation [3] and asked them to test all infants presenting at hospital with pertussis-like symptoms. ‘Typical’ pertussis presentation was defined either by a presence of apnoea; or by a cough associated with at least one of paroxysms, whoop or post-tussive vomiting. When physicians suspected pertussis even though some typical symptoms were missing, pertussis was considered as ‘atypical’.

We identified all infants attending the hospital who were tested for pertussis and invited their parents to participate in the study. When required by the local ethical committee, parents or legal guardians were requested to provide an informed consent.

**Figure 1:**
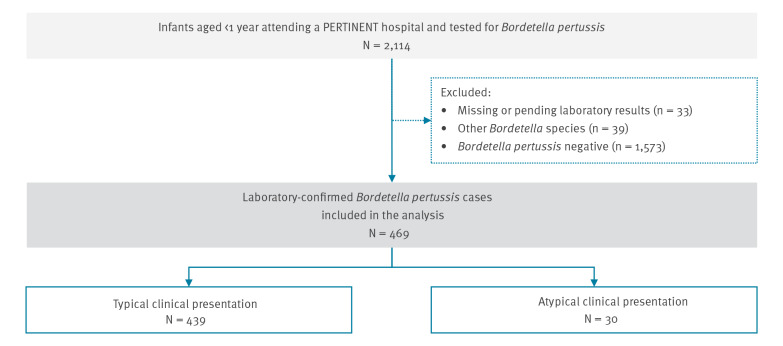
Flowchart of hospitalised infants’ (< 1 year) inclusion in or exclusion from the PERTINENT study, Europe, 1 December 2015–31 December 2018 (n = 2,144 hospitalised infants)

We excluded all patients with missing or pending laboratory results, testing positive to other *Bordetella* species than *B. pertussis* or whose legal guardian was unwilling to participate or unable to communicate and give consent. All laboratory-confirmed *B. pertussis* cases aged less than 1 year at the time of hospitalisation were included in the study (Figure 1).

PERTINENT: Pertussis in Infants European Network.

### Definitions

We defined a laboratory-confirmed *B. pertussis* case as an infant attending one of the participating hospitals (irrespective of the length of stay), aged less than 1 year and testing positive for *B. pertussis* by PCR (DNA detection of *B. pertussis* using PCR or real-time PCR in a nasopharyngeal aspirate or swab) or culture (isolation of *B. pertussis* from the prior-mentioned clinical specimen) regardless of the clinical criteria.

We defined a severe case as a case admitted to the intensive care unit (ICU).

We defined a likely source of infection as a person with a cough who had contact with the case in the 7 to 20 days before the date of symptom onset of the case.

For each pertussis vaccine dose, we defined an infant as vaccinated if she/he had received the dose of interest > 14 days before symptom onset. Unvaccinated infants were those who had not received any dose or who had received the first dose ≤ 14 days before symptom onset.

### Laboratory methods

To ensure an accurate identification of the *Bordetella* species, the PERTINENT laboratory guidelines recommend a diagnostic algorithm for DNA detection of *Bordetella* including a series of three PCRs: a triplex real-time PCR targeting IS*481* gene (in *B. pertussis*, *holmesii* and some *bronchiseptica* strains), pIS*1001* (*B. parapertussis*) and *RNase* P as the human internal control; followed by two confirmatory singleplex tests targeting *ptxA*-Pr (*B. pertussis*) and hIS*1001* (*B. holmesii*) genes [17].

### Data collection

Using a standard questionnaire, all sites collected a common set of information: demographic, epidemiological, clinical, laboratory data, vaccination status of the infant and household members, risk and protective factors and suspected source of infection. The list of potential sources of infection included close relatives and caregivers. Each study site translated the questionnaire available in English in its country’s language. Data were collected through review of clinical case notes, extraction from patient registries or, if not available, interviews with parents or legal guardians.

### Denominators

The denominator was the estimated population of infants aged less than 1 year likely to receive care at hospitals participating in the study. The methods to estimate the hospitals’ catchment population varied across sites (Table 1). Throughout the study period, we adjusted the catchment population to the loss of the four Norwegian hospitals.

### Analysis

We described cases by age, clinical presentations, risk and protective factors, severity. We used median for continuous variables (age in months, gestational age and weight at birth) and frequencies for categorical variables. We estimated the incidence by site and year. In four sites, the protocol was implemented during 2016 and we estimated 2016 incidence *prorata temporis*. We calculated incidence rate ratios (IRR) for 2018 compared with 2017.

We used Fisher’s exact test to compare clinical signs and symptoms by age group and characteristics of cases admitted to ICU with those not admitted to ICU.

### Ethical statement

The planning, conduct and reporting of the study was in line with the Declaration of Helsinki [18]. Ethical approval was not needed in Navarra as the PERTINENT study came under the umbrella of the mandatory surveillance system. Other study sites sought ethical approval from a review board according to country-specific regulations (Catalonia: PIC-31–16, Czech Republic: SZU/05992/2019, France: n°449199 v 1, Ireland: REC reference number 16.058 and Gen/499/16, Italy: Bambino Gesù Children's Hospital Ethical Committee: protocol n. 1064_OPBG_2016, Norway: REC register number 2015/956).

## Results

### Description of cases

Of the 2,114 infants tested for *B. pertussis*, 2,081 had laboratory results available (98%) (Figure 1). We excluded 39 cases caused by other *Bordetella* species (2%). A total of 469 infants were positive for *B. pertussis* (23%) including one co-infection with *B. pertussis* and *parapertussis*. The number of pertussis cases by site ranged from seven in Norway to 199 cases in France (Table 1).

Among the 469 laboratory-confirmed *B. pertussis* cases, information on the sample type was available for 448, with 368 (82%) having had a nasopharyngeal aspirate, 102 (23%) a nasopharyngeal swab and 22 (5%) both. Of all 469 confirmed cases, 465 had a PCR (99%) and 255 a culture done (54%). All PCRs and 108 of 211 culture results available (51%) were positive to *B. pertussis* (Table 2).

**Table 2:** Hospitalised *Bordetella pertussis* cases aged < 1 year by age group, sex, laboratory components, clinical presentation, most likely source of infection and severity criteria, PERTINENT study, Europe, 1 December 2015–31 December 2018 (n = 469 cases)

Characteristic		Cases (all < 1 year old) (n = 469)		Cases 0–3 months old (n = 354)		Cases 4–11 months old (n = 115)		p value
		Number	%	Number	%	Number	%	
**Demographic**								
Sex (n = 469)	Female	222	47.3	165	46.6	57	49.6	0.593
	Male	247	52.7	189	53.4	58	50.4	
**Laboratory components**								
Nasopharyngeal specimen collection (n = 448)	Aspirate only	346	77.2	252	75.4	94	82.5	0.321
	Swab only	80	17.9	64	19.2	16	14.0	
	Both	22	4.9	18	5.4	4	3.5	
PCR (n = 465)	Positive	465	100.0	351	100.0	114	100.0	NA
	Negative	0	0.0	0	0.0	0	0.0	
Culture (n = 211)^a^	Positive	108	51.2	89	51.7	19	48.7	0.859
	Negative	103	48.8	83	48.3	20	51.3	
**Clinical presentation**								
Cough (n = 469)	Yes	456	97.2	342	96.6	114	99.1	0.202
	No	13	2.8	12	3.4	1	0.9	
Cough with paroxysms (n = 456)	Yes	393	86.2	298	87.1	95	83.3	0.347
	No	63	13.8	44	12.9	19	16.7	
Whooping cough (n = 269)	Yes	122	45.4	91	47.2	31	40.8	0.415
	No	147	54.6	102	52.8	45	59.2	
Post-tussive vomiting (n = 449)	Yes	219	48.8	164	48.7	55	49.1	1.000
	No	230	51.2	173	51.3	57	50.9	
Apnoea (n = 466)	Yes	235	50.4	193	55.0	42	36.5	0.001
	No	231	49.6	158	45.0	73	63.5	
Cyanosis (n = 467)	Yes	239	51.2	192	54.5	47	40.9	0.013
	No	228	48.8	160	45.5	68	59.1	
Epidemiological link (n = 457)	Yes	167	36.5	124	35.8	43	38.7	0.651
	No	290	63.5	222	64.2	68	61.3	
Diagnosis by a clinician (n = 469)	Yes	368	78.5	275	77.7	93	80.9	0.516
	No	101	21.5	79	22.3	22	19.1	
**Reported source of infection**								
Mother (n = 424)	Yes	106	25.0	88	27.5	18	17.3	0.038
	No	318	75.0	232	72.5	86	82.7	
Father (n = 419)	Yes	82	19.6	66	21.0	16	15.4	0.255
	No	337	80.4	249	79.0	88	84.6	
Sibling (n = 416)	Yes	128	30.8	102	32.8	26	24.8	0.143
	No	288	69.2	209	67.2	79	75.2	
Grandparents (n = 409)	Yes	41	10.0	33	10.8	8	7.7	0.451
	No	368	90.0	272	89.2	96	92.3	
Caregiver (n = 240)	Yes	0	0.0	0	0.0	0	0.0	NA
	No	240	100.0	175	100.0	65	100.0	
**Severity criteria**								
Death (n = 466)	Yes	5	1.1	5	1.4	0	0.0	0.340
	No	461	98.9	346	98.6	115	100.0	
ICU (n = 330)	Yes	83	25.2	80	31.5	3	3.9	0.000
	No	247	74.8	174	68.5	73	96.1	
ECMO (n = 336)	Yes	7	2.1	7	2.7	0	0.0	0.360
	No	329	97.9	251	97.3	78	100.0	
Pneumonia (n = 327)	Yes	14	4.3	12	4.8	2	2.7	0.745
	No	313	95.7	240	95.2	73	97.3	
Encephalopathy (n = 327)	Yes	3	0.9	3	1.2	0	0.0	1.000
	No	324	99.1	249	98.8	75	100.0	
Seizure (n = 328)	Yes	10	3.0	8	3.2	2	2.7	1.000
	No	318	97.0	245	96.8	73	97.3	
Eating difficulties (n = 269)	Yes	62	23.0	50	25.4	12	16.7	0.144
	No	207	77.0	147	74.6	60	83.3	
Kidney failure (n = 267)	Yes	4	1.5	4	2.0	0	0.0	0.576
	No	263	98.5	192	98.0	71	100.0	
Dehydration (n = 300)	Yes	12	4.0	11	4.8	1	1.4	0.305
	No	288	96.0	216	95.2	72	98.6	

ECMO: Extracorporeal membrane oxygenation; ICU: intensive care unit; PERTINENT: Pertussis in Infants European Network.

^a ^Cultures were done for 255 cases, but the results were only available for 211 cases.

Number of cases presented for the different characteristics are those with information available.

Of the 469 cases, 247 were males (53%). The median age was 2.5 months (range: 0–11.6; interquartile range (IQR): 2.5). The median weight at birth was 3,280 g (range: 700–4,925; IQR: 720). The median gestational duration was 39 weeks (range: 25–42; IQR: 2).

**Figure 2:**
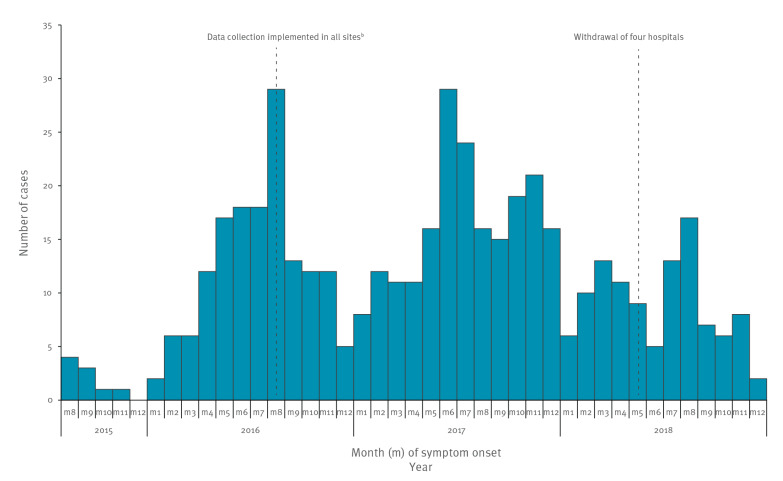
Hospitalised *Bordetella pertussis* cases aged < 1 year by month of symptom onset, PERTINENT study, Europe, 1 December 2015–31 December 2018 (n = 464 cases^a^)

The number of reported cases by month of symptom onset (Figure 2) was highest in August 2016 (n = 29) and in June 2017 (n = 29). Excluding the first months of progressive surveillance implementation, less cases were observed at the transition between years, such as December 2016 (n = 5), January 2018 (n = 6) and December 2018 (n = 2), but not always, as for example June 2018 (n = 5). 

PERTINENT: Pertussis in Infants European Network.

^a^ Five patients with missing date of symptom onset are not included in the figure.

^b ^Data collection started in 2015, but was fully operational in all sites in August 2016 only.

**Figure 3:**
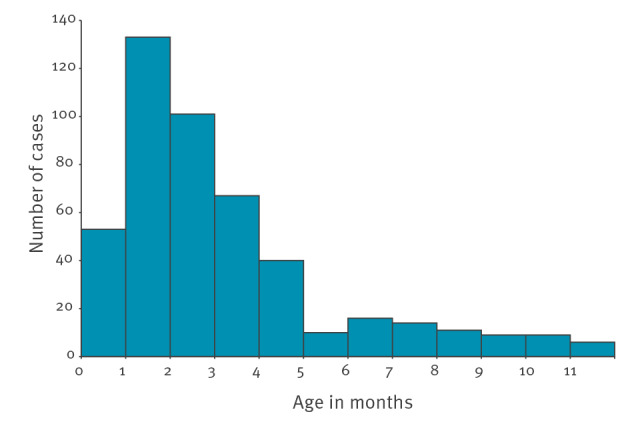
Hospitalised *Bordetella pertussis* cases aged < 1 year by age in months, PERTINENT study, Europe, 1 December 2015–31 December 2018 (n = 469 cases)

The highest number of cases was reported in the age group 0–2 months (n = 287), with a peak in the second month of life (n = 133), then the number decreased by age in months (Figure 3).

PERTINENT: Pertussis in Infants European Network.

Of the 469 cases, 456 (97%) had a cough, among whom 86% had paroxysm. For those who had cough and additional information on post-tussive vomiting (n = 449) and whoop (n = 269), 49% had post-tussive vomiting and 45% whoop. For cases with available data on apnoea (n = 466), 235 (50%) had apnoeic episodes (Table 2), including six cases without cough. Pertussis was typical in 439 cases (94%) (Figure 1). Thirty cases had an atypical pertussis clinical presentation (6%), among whom 20 had a cough only, three had cough and cyanosis only, two had a cyanosis only and five had documented absence of all pertussis-like symptoms. Four of these atypical pertussis cases had at least one missing clinical information.

Among 354 cases aged 0–3 months, 250 (71%) had either apnoea or cyanosis compared with 62 (54%) among 115 cases aged 4–11 months (p = 0.001) (Table 2).

Clinical presentation did not differ between vaccinated and not vaccinated infants (data not shown). Of all cases, 290 (62%) were eligible for vaccination. Of the 273 with information on vaccination status, 106 (39%) were vaccinated: 59 had received one dose, 39 two doses and eight three doses of pertussis vaccine. Of the 30 atypical cases, 18 were eligible for vaccination and, of the 17 with related data, about half (n = 9) had received one or two doses.

### Potential source of infection

Information on the potential source of infection was available for 441 cases (94%). Infants’ sibling was mentioned for 128 cases (31%), the mother for 106 cases (25%), the father for 82 cases (20%) and the grandparents for 41 cases (10%) (Table 2).

### Severity

Five (1%) of 466 cases with information died. They were aged from 2 weeks to 10 weeks and not vaccinated against pertussis. In two of the five cases, the mother was vaccinated 5 to 10 years earlier. For one case, the mother was vaccinated 5 days before delivery. Vaccination status of the mother was unknown for the remaining two cases.

Eighty-three cases (25%) were admitted to ICU, including seven cases treated with extracorporeal membrane oxygenation (ECMO) and the five infants who died (Table 2). Among infants aged 0–3 months, 80 (32%) were admitted to ICU compared with three (4%) among infants aged 4–11 months (p < 0.001) (Table 2). The median age in months was 1.4 (range: 0–4.6; IQR: 1.3) in cases who required ICU admission and 2.8 (range: 0.1–11.6; IQR: 2.7) in those who did not (p < 0.001). Of ICU cases, 66 (80%) had either apnoea or cyanosis compared with 160 (65%) in non-ICU cases (p = 0.014). The proportion of cases with comorbidities was similar between the two groups. Among cases with available gestational week, 21 of the 82 ICU cases (26%) and 26 of 247 non-ICU cases (11%) were preterm infants born before gestational week 37 (p = 0.002) (Table 3).

**Table 3:** Hospitalised *Bordetella pertussis* cases aged < 1 year by ICU admission, clinical presentation, comorbidities and risk/protective factors, PERTINENT study, Europe, 1 December 2015–31 December 2018 (n = 330 cases^a^)

Characteristic		Admitted to the ICU (n = 83)		Not admitted to the ICU (n = 247)		p value
		Number	%	Number	%	
**Clinical presentation**						
Cough (n = 330)	Yes	83	100.0	238	96.4	0.119
	No	0	0.0	9	3.6	
Paroxysms among those with cough (n = 321)	Yes	73	88.0	203	85.3	0.714
	No	10	12.0	35	14.7	
Whoop (n = 252)	Yes	17	43.6	101	47.4	0.728
	No	22	56.4	112	52.6	
Post-tussive vomiting (n = 320)	Yes	45	54.2	110	46.4	0.251
	No	38	45.8	127	53.6	
Apnoea (n = 328)	Yes	55	66.3	128	52.2	0.030
	No	28	33.7	117	47.8	
Cyanosis (n = 329)	Yes	59	71.1	106	43.1	0.000
	No	24	28.9	140	56.9	
**Comorbidities**						
Cardiovascular (n = 330)	Yes	4	4.8	6	2.4	0.278
	No	79	95.2	241	97.6	
Respiratory (n = 330)	Yes	2	2.4	4	1.6	0.644
	No	81	97.6	243	98.4	
Immunodeficiencies (n = 129)	Yes	0	0.0	0	0.0	NA
	No	57	100.0	72	100.0	
**Risk/protective factors**						
Breastfeeding (n = 327)	Yes	50	62.5	166	67.2	0.497
	No	30	37.5	81	32.8	
Premature 37 weeks (n = 329)	Yes	21	25.6	26	10.5	0.002
	No	61	74.4	221	89.5	

ICU: intensive care unit; PERTINENT: Pertussis in Infants European Network.

^a^ 330 cases of 469 had information on ICU or non-ICU admission.

Numbers of cases with available information are presented.

Among infants eligible for vaccination, seven of the 25 ICU cases (28%) and 65 of the 163 non-ICU cases (40%) were vaccinated with at least one dose of pertussis vaccine (p = 0.279). None of the ICU cases and 35 of the non-ICU cases (21%) had received two or more doses of pertussis vaccine (p = 0.005).

### Incidence by site

Incidence rate ratios between 2018 and 2017 were 1.43 in Czech Republic (p = 0.468), 0.25 in Catalonia (p = 0.002), 0.71 in France (p = 0.0335), 0.14 in Ireland (p = 0.002), 0.63 in Italy (p = 0.053), 0.21 in Navarra (p = 0.148) and zero in Norway (Table 1).

### Other *Bordetella* species

Five of the seven sites provided information on other *Bordetella* species. We identified 40 respiratory infections caused by other *Bordetella* species, including 17 *B. parapertussis* (cases aged 6 to 51 weeks) and four *B. holmesii* (cases aged 7 to 14 weeks). Of the 17 *B. parapertussis* cases, two did not have typical pertussis-like symptoms but only cough and three of 12 *B. parapertussis* cases with information were admitted to ICU. Patients infected with *B. holmesii* had pertussis-like symptoms and did not require ICU admission. Among infants eligible for vaccination, eight of 13 *B. parapertussis* cases and two of three *B. holmesii* cases were vaccinated with at least one dose of pertussis vaccine.

## Discussion

Over the three pilot years of the PERTINENT active hospital-based surveillance system, we identified 469 laboratory-confirmed *B. pertussis* cases. We observed a higher incidence in 2017 and a decrease in 2018 in all but one study site. In 2016 and 2017, most of the cases were reported during summer. One of four pertussis laboratory-confirmed hospitalised cases was admitted to ICU. The highest proportion of ICU cases was in infants aged 0–3 months. Five infants died, all were unvaccinated: either too young to be vaccinated or in the month of life targeted for the first dose of the primary schedule.

Despite standard protocols proposed, the PERTINENT pilot surveillance still has limits to consider when examining the findings. Different methods were used by study sites for estimating hospital catchment population. This may have biased the measured incidence and made annual comparison between sites difficult. However, those methods did not change over time and allowed to compute and compare incidence by sites. During the pilot phase, one country had to decrease the number of participating hospitals and we adjusted the incidence denominator accordingly.

Among the five sites that provided information on other *Bordetella* species, the proportion of patients with other *Bordetella* species among those testing positive for any *Bordetella* species was 11% (40/357) on average, ranging from 0 of 25 to 5 of 18. Two sites were not able to differentiate *B. pertussis* from the other *Bordetella* species that may induce a similar respiratory presentation upon infection. This likely decreased the specificity of the laboratory testing and increased reported incidences.

PERTINENT laboratories did not so far sequence *B. pertussis* isolates, which prevented detection of changes in the pathogen. Isolates were stored in optimal conditions to be analysed later on.

Severity of the disease remains difficult to interpret because of different severity ascertainment and clinical practices by country and by hospital. Hospitalised cases are usually severe pertussis cases, however, the probability of being hospitalised for pertussis-like symptoms is heterogeneous across sites due to different referral to hospital practices. In France, any infant aged less than 3 months with pertussis-like symptoms will be admitted regardless of the severity of the disease. In contrast, a large proportion of Norwegian infants will first attend the ‘out-of-office emergency primary care’ services (legevakt) and only severe cases will be transferred to hospitals. This likely affected the comparison of clinical signs and incidence rate of hospitalised severe cases between sites.

The likely source of infection was ascertained by family interviews, which may require caution in interpreting the results. Caregivers were not reported as probable sources for any of the reported pertussis cases. As parents self-reported the likely source of infection, they may have better remembered signs of coughing in the household members than among the infant’s caregivers. Additional questions, laboratory confirmation in suspected sources of infection, different study designs with increased data completeness are needed to identify source of infection.

Despite using a standard protocol in all sites, data completeness still needs improvement. In severity variables, completeness ranged from 57% (267/469) to 72% (336/469), except for death ascertainment with a completeness > 99% (Table 2).

Taking the above limitations into account, our results suggest a decrease in 2018 in pertussis incidence compared with 2017, across all sites except for the Czech Republic site where incidence remained stable. The low number of study sites does not allow to extrapolate results to national nor EU/EEA level. However, incidences reported to the European Surveillance System (TESSy) by five of the six countries involved in PERTINENT also suggest a decrease. Incidences reported for hospitalised and non-hospitalised cases in 2018 ranged from 42.9 to 85.3 per 100,000 infants in all infants aged less than 1 year, as compared with a range of 12.9 to 87.6 per 100,000 infants in PERTINENT for hospitalised infants only (excluding Norway where zero cases were observed) suggesting a better sensitivity of the PERTINENT data.

We suggest that the summer peak observed in reported cases might possibly reflect the seasonality of the disease. It is unlikely that this was due to improved diagnosis in those months as the PERTINENT surveillance system was stable over time. An increase in pertussis cases during summer was previously reported. In the Netherlands where pertussis is a statutory notifiable disease, the annual peak incidence of notifications for all age groups (0–4; 5–12; 13–18 and 19–99 years) between 1996 and 2006 was in August [19]. More recently, using the notifiable infectious disease reporting system in China from January 2004 to May 2018, Wang suggested a seasonality in pertussis cases and a summer peak with a maximum in August [20].

Thirty cases did not have a typical pertussis clinical presentation including four with at least one clinical sign not documented and five cases with documented absence of all pertussis-like symptoms (reason for hospitalisation unknown). The EU case definition for pertussis was revised in June 2018 [21] to draw attention on atypical symptoms in adults, adolescents or vaccinated children. Our results may suggest the existence of atypical pertussis in infants [3] and highlight the need to raise clinicians’ awareness about possible under-diagnosis of pertussis in that age group. The World Health Organization and United States Centers for Disease Control and Prevention pertussis case definitions do not include isolated apnoea or cyanosis in the clinical criteria for pertussis surveillance. In our study, pertussis cases aged less than 3 months were more likely to present with apnoea or cyanosis, which supports the inclusion of these clinical criteria in the pertussis case definition.

Our results suggest that cases admitted to ICU were younger and less vaccinated than non-ICU cases. In the PERTINENT hospital-based sentinel network, four pertussis deaths were reported in 2017 across the seven study sites. As pertussis cases identified in the study were followed up during hospitalisation, we expect no under-reporting of deaths. Among pertussis cases aged less than 1 year reported to TESSy, there were three pertussis deaths in 2017 across the 29 EU/EEA reporting countries [2]. This may suggest that detection of pertussis hospital death is more sensitive in the PERTINENT system. As described in other systems, deaths may be under-ascertained in routine hospital based surveillance in EU/EEA countries [22,23].

The most likely source of infection reported by the parents was firstly the patient’s siblings followed by the mother. Recent studies have also shown an increased risk of transmission to siblings of primary cases [24] and a shift in the source of infection from the mother to the siblings [25]. This may be a consequence of vaccinating the mother either before, during or after pregnancy (cocooning strategy), therefore preventing transmission to infants.

*Bordetella* species can be isolated from both nasopharyngeal swabs or aspirates but a 15% gain in the isolation rate can be obtained by using aspirates in neonates and infants [26]. In our study, we reached a good quality of specimen collection with a high proportion of nasopharyngeal aspirates (82%). We identified 40 infections caused by other *Bordetella* species, including four with *B. holmesii*, which is rarely isolated in infants [27]. Even though other *Bordetella* species are not directly targeted by pertussis-containing vaccine, the later may also induce some cross-immunity for specific *Bordetella* species [28].

### Conclusions

This pilot project shows that enhanced pertussis surveillance in Europe is possible. The generic protocol presented some challenges and efforts by all partners were needed to improve data quality and laboratory procedures but we believe this allowed to pool sites’ data to better describe hospitalised laboratory-confirmed pertussis cases, as these were recruited using the same criteria across six EU/EEA countries. However, a larger sustained project is needed with additional countries to ensure representativeness in Europe and a particular emphasis on harmonisation of laboratory methods. In the future, this surveillance network should allow monitoring emergence of atypical pertussis presentation, identifying upcoming pertussis epidemic cycles and comparing incidence over time in Europe according to immunisation strategies. It will also allow measuring the effectiveness of infants’ and mothers’ vaccination. 

## PERTINENT group

**Epiconcept, France: **Lore Merdrignac, Epiconcept; Camelia Savulescu, Epiconcept; Marta Valenciano, Epiconcept; Alain Moren, Epiconcept.

**Czech Republic: **Pavla Křížová, National Institute of Public Health, Prague; Kateřina Fabiánová, National Institute of Public Health, Prague; Jana Zavadilová, National Institute of Public Health, Prague; Zuzana Blechová, University Hospital Na Bulovce, Prague; Květa Bláhová, University Hospital Motol, Prague; Pavel Kosina, University Hospital, Hradec Králové; Josef Sýkora, University Hospital, Pilsen; Alena Holčíková, University Hospital, Brno; Petr Širůček, University Hospital, Ostrava.

**France: **Daniel Lévy-Brühl, Santé Publique France, Saint-Maurice; Fatima Aït El Belghiti, Santé Publique France, Saint-Maurice; Adèle Grembombo, Santé Publique France, Saint-Maurice; Sophie Guillot, Institut Pasteur, Paris; Sylvain Brisse, Institut Pasteur, Paris; Julie Toubiana, Institut Pasteur, Paris.

**Ireland: **Suzanne Cotter, HSE-Health Protection Surveillance Centre, Dublin; Jane Murphy, Temple Street Children’s University Hospital, Dublin; Robert Cunney, Temple Street Children’s University Hospital, Dublin; Norma O’Shaughnessy, Temple Street Children’s University Hospital, Dublin; Adele Habington, Our Lady’s Children’s hospital Crumlin, Dublin; Niamh O’Sullivan, Our Lady’s Children’s hospital Crumlin, Dublin.

**Italy: **Elisabetta Pandolfi, Bambino Gesù Children Hospital, Rome; Alberto E Tozzi, Bambino Gesù Children Hospital, Rome; Caterina Rizzo, Bambino Gesù Children Hospital, Rome; Luisa Russo, Bambino Gesù Children Hospital, Rome; Ilaria Campagna, Bambino Gesù Children Hospital, Rome; Francesco Gesualdo, Bambino Gesù Children Hospital, Rome; Sara Ciampini, Bambino Gesù Children Hospital, Rome; Valentina Annarosa Ferro, Bambino Gesù Children Hospital, Rome; Elena Boccuzzi, Bambino Gesù Children Hospital, Rome.

**Norway: **Elmira Flem, Norwegian Institute of Public Health, Oslo; Håkon Bøås, Norwegian Institute of Public Health, Oslo; Terese Bekkevold, Norwegian Institute of Public Health, Oslo; Liliana Vazquez Fernandez, Norwegian Institute of Public Health, Oslo.

**Catalonia, Spain: **Carmen Muñoz-Almagro, Instituto de Recerca Pediatrica Hospital Sant Joan de Deu, Barcelona; Universitat Internacional de Catalunya and CIBER of Epidemiology and Public Health CIBERESP; Cristina Esteva, Instituto de Recerca Pediatrica Hospital Sant Joan de Deu, Barcelona; CIBER of Epidemiology and Public Health CIBERESP; Miguel Lanaspa Perez, Instituto de Recerca Pediatrica Hospital Sant Joan de Deu, Barcelona; Mireia Jané, Public Health Agency of Catalonia, Barcelona; CIBER of Epidemiology and Public Health CIBERESP; University of Barcelona; Gloria Carmona, Public Health Agency of Catalonia, Barcelona; Lesly Acosta, Universitat Politècnica de Catalunya - BarcelonaTech (UPC), Public Health Agency of Catalonia, Barcelona; Yolanda Jordan Garcia, Instituto de Recerca Pediatrica Hospital Sant Joan de Deu, Barcelona; CIBER of Epidemiology and Public Health CIBERESP.

**Navarra, Spain: **Manuel García Cenoz, Instituto de Salud Pública de Navarra, IdiSNA – Navarre Institute for Health Research, Pamplona; Ana Navascués, Complejo Hospitalario de Navarra, Pamplona; Leticia Fernandino Zubieta, Instituto de Salud Pública de Navarra, IdiSNA – Navarre Institute for Health Research, Pamplona; Jesús Castilla, Instituto de Salud Pública de Navarra, IdiSNA - Navarre Institute for Health Research, Pamplona.

**Sentinelles, France: **Thomas Hanslik, Sorbonne Université, INSERM, Institut Pierre Louis d’Epidémiologie et de Santé Publique (IPLESP UMRS 1136), Paris.

**ECDC: **Sabrina Bacci, European Centre for Disease Prevention and Control, Stockholm, Sweden; Gianfranco Spiteri, European Centre for Disease Prevention and Control, Stockholm, Sweden.

## Supplementary Data

71000 Supplementary Material
