# Supplementary material for: Incidence and severity of pertussis hospitalisations in infants aged less than 1 year in 37 hospitals of six EU/EEA countries, results of PERTINENT sentinel pilot surveillance system, December 2015 to December 2018
Source: Euro Surveill. 2021 Jan 28;26(4):1900762. doi: 10.2807/1560-7917.ES.2021.26.4.1900762 (PMC7848786; doi:10.2807/1560-7917.ES.2021.26.4.1900762)
Supplement: Supplementary Material [file 1900762_Supplement.pdf]

This supplementary material is hosted by Eurosurveillance as supporting information alongside the article [Incidence and severity of pertussis hospitalisations in infants aged less than 1 year in 37 hospitals of six EU/EEA countries, results of PERTINENT sentinel pilot surveillance system, December 2015 to December 2018.], on behalf of the authors, who remain responsible for the accuracy and appropriateness of the content. The same standards for ethics, copyright, attributions and permissions as for the article apply. Supplements are not edited by Eurosurveillance and the journal is not responsible for the maintenance of any links or email addresses provided therein.

Table S4. Vaccination recommendations, introduction year and vaccine coverage by PERTINENT study sites, PERTINENT, 1st December 2015 - 31st December 2018

**Study sites**		**Czech Republic**	**France**	**Ireland**	**Italy**	**Spain, Catalonia**	**Spain, Navarra**	**Norway**
Primary schedule in infants <1 year	Year introduction	2018	2013	1995	1995	2016	2016	1998
	Schedule	3, 5, 11-13 months	2, 4, 11 months	2, 4, 6 months	3, 5, 11 months	2, 4, 11 months	2, 4, 11 months	3, 5, 12 months
	VC % in 2016	94.4%	95.8% at 24 mo	90% at 24 mo	96.8% at 24 mo	94.0%	96.8%	96% at 2 yo
Pregnancy	Year introduction	2016	no	2013	2017	2014	2015	no
	VC %	*Not determined*	NA	49.9% in 2017-2018	*Not determined*	79% in 2017	89% in 2017	NA
Cocooning	Year introduction	no	2004	2013	no	no	no	no
	VC %	NA	<50% in 2004-2015	*Not determined*	NA	NA	NA	NA
Booster doses	Schedule	5-6 yo<br>10-11 yo	6 yo<br>11-13 yo	4-5 yo<br>12-13 yo	6 yo<br>12-18 yo	6 yo	6 yo	7 yo<br>15 yo
Adults	Schedule	1 dose in life	1 dose every 10 years	no	1 dose every 10 years	no	no	1 dose every 10 years
